# A dynamic multi-branch EEG decoding network for motor imagery classification with preliminary clinical validation

**DOI:** 10.3389/fnins.2026.1918072

**Published:** 2026-09-10

**Authors:** Jingxin Cai, Mengyao Gao, Guangyu Li, Xiaomeng Zhao, Chenglin Xu, Lei Chen, Fangzhou Xu, Fulei Hu

**Affiliations:** 1 School of Medical Information Engineering, Shandong University of Traditional Chinese Medicine, Jinan, Shandong, China; 2 Affiliated Hospital of Shandong University of Traditional Chinese Medicine, Jinan, Shandong, China; 3 International School for Optoelectronic Engineering, Qilu University of Technology (Shandong Academy of Sciences), Jinan, Shandong, China; 4 Jinan Engineering Laboratory of Human-Machine Intelligent Cooperation, Jinan, Shandong, China; 5 Rehabilitation and Physical Therapy Department, Affiliated Hospital of Shandong University of Traditional Chinese Medicine, Jinan, Shandong, China

**Keywords:** motor imagery, brain–computer interface, EEG decoding, trial-conditioned dynamic fusion, learnable time-frequency representation, spinal cord injury

## Abstract

Motor imagery electroencephalography (MI-EEG) decoding remains challenging because of the low signal-to-noise ratio, non-stationarity, and inter-subject variability of EEG signals. This study proposes a dynamic multi-branch EEG decoding network (DMB-EDN) that jointly models temporal dynamics, learnable time-frequency patterns, and rhythm-specific spectral information. DMB-EDN combines a learnable Gabor-based time-frequency representation with physiologically guided rhythm modeling and employs trial-conditioned dynamic fusion to estimate the contribution of each branch separately for each EEG trial. This design enables adaptive coordination of complementary data-driven and physiology-guided representations. The proposed method was evaluated on the BCI Competition IV 2a dataset, the High Gamma Dataset, and a self-collected spinal cord injury (SCI) dataset. Under subject-specific evaluation, DMB-EDN achieved an average accuracy of 96.41% and a kappa of 0.952 on BCI Competition IV 2a. On the High Gamma Dataset, it achieved performance comparable to the strongest baseline under near-saturated conditions. Under leave-one-subject-out evaluation on the SCI dataset, DMB-EDN obtained an accuracy of 85.00% and a kappa of 0.700, providing preliminary evidence of improved offline cross-subject decoding. Ablation experiments confirmed the complementary contributions of the three representation branches and trial-conditioned fusion, while fusion-weight analysis revealed systematic class- and oscillation-related variations. These results demonstrate the effectiveness of DMB-EDN for EEG decoding, although validation on larger multicenter cohorts and prospective online BCI systems remains necessary.

## 1 Introduction

Brain–computer interface (BCI) establish a direct communication pathway between the human brain and external devices without relying on conventional neuromuscular output (Wolpaw et al., 2002). Among non-invasive BCI paradigms, motor imagery electroencephalography (MI-EEG) has received considerable attention because imagined movements can modulate sensorimotor rhythms without requiring overt motor execution. These modulations are commonly manifested as event-related desynchronization and synchronization over the sensorimotor cortex, providing neurophysiological signals for assistive control, neuroprostheses, and rehabilitation training (Pfurtscheller and Lopes da Silva, 1999; Ang and Guan, 2017). MI-based BCIs are particularly relevant to individuals with motor impairments, including patients with stroke or spinal cord injury, for whom decoding movement intention from EEG may facilitate communication and motor rehabilitation (Ang and Guan, 2017; Opsommer et al., 2020).

Despite this potential, reliable MI-EEG decoding remains challenging. EEG signals have a low signal-to-noise ratio, limited spatial resolution, strong non-stationarity, and considerable susceptibility to physiological and environmental artifacts (Padfield et al., 2019). Moreover, discriminative EEG patterns may vary substantially across subjects, sessions, trials, and even different periods within the same trial, resulting in considerable intra-subject and inter-subject variability (Saha and Baumert, 2020). Recent systematic evaluations have further identified limited training data, inconsistent experimental protocols, and insufficiently controlled feature fusion as important obstacles to fair and reliable MI-EEG decoding (Wang et al., 2024). These characteristics make it difficult for a fixed representation or uniform integration strategy to maintain stable performance across both public benchmark datasets and heterogeneous clinical recordings.

Traditional MI-EEG decoding methods generally rely on handcrafted feature extraction followed by shallow classifiers. Common spatial pattern (CSP) learns spatial filters that maximize the variance difference between motor imagery classes (Ramoser et al., 2000), while its variants and related spatial-filtering methods improve single-trial EEG discrimination through subject-specific spatial optimization (Blankertz et al., 2008). Filter-bank common spatial pattern further decomposes EEG signals into multiple predefined frequency intervals and selects discriminative band-specific CSP features (Ang et al., 2012). Although these approaches are computationally efficient and provide a degree of neurophysiological interpretability, their performance depends strongly on manually specified frequency bands, temporal intervals, feature-selection criteria, and classifiers. Consequently, they may not fully capture the complex nonlinear interactions among temporal, spatial, and spectral EEG characteristics.

Deep neural networks provide an alternative by learning hierarchical representations directly from EEG signals. ShallowConvNet and DeepConvNet demonstrated that convolutional neural networks can jointly learn temporal and spatial filters from minimally processed EEG (Schirrmeister et al., 2017), while EEGNet introduced depthwise and separable convolutions to construct a compact architecture applicable to different EEG paradigms (Lawhern et al., 2018). EEG-Inception subsequently employed parallel temporal convolutions with different receptive fields to capture multi-scale temporal patterns (Zhang et al., 2021). FBCNet incorporated filter-bank representations, spatial convolution, and variance-based temporal aggregation to learn frequency-aware and spatially discriminative features (Mane et al., 2020). These approaches substantially reduce dependence on handcrafted feature engineering and improve temporal, spatial, or frequency-aware representation learning. Nevertheless, compact single-path models tend to learn entangled representations, whereas multi-scale and filter-bank models generally integrate their views through predefined architectural pathways.

Temporal convolutional and attention-based methods have further improved sequential EEG modeling. EEG-TCNet combines compact convolutional encoding with a temporal convolutional network to capture longer-range temporal dependencies while maintaining a relatively small computational footprint (Ingolfsson et al., 2020). TCNet-Fusion enriches temporal representations by fusing features extracted from different levels of a temporal convolutional architecture (Musallam et al., 2021). ATCNet combines convolutional sliding windows, multi-head self-attention, and temporal convolution to emphasize informative temporal intervals and model local and long-range dependencies (Altaheri et al., 2023). EEG Conformer integrates convolution-based local feature extraction with Transformer-based global dependency modeling (Song et al., 2023), while LMDA-Net introduces channel- and depth-attention modules for lightweight multidimensional EEG feature enhancement (Miao et al., 2023). Although these methods provide effective temporal modeling and adaptive feature recalibration, their attention mechanisms mainly operate over temporal windows, channels, tokens, or latent feature dimensions. They do not necessarily determine the relative contribution of semantically distinct temporal, learnable time-frequency, and rhythm-specific representations for each EEG trial.

Multi-branch, multi-scale, and multi-domain architectures have therefore become important directions in MI-EEG decoding. MBEEGSE uses parallel EEGNet branches with different configurations and squeeze-and-excitation blocks to improve representation diversity and channel-wise recalibration (Altuwaijri et al., 2022). MSVTNet combines multi-scale convolutional encoding with Transformer-based interaction modeling to capture local spatiotemporal characteristics, cross-scale relationships, and global dependencies (Liu et al., 2024). MT-MBCNN explicitly constructs spatial–temporal–spectral representations through a multi-branch and multi-task framework (Cai et al., 2024), whereas MSFF-SENet integrates multi-scale spatiotemporal features with power-spectral-density representations through attention-based feature fusion (Gao et al., 2024). These studies demonstrate that multi-branch and multi-domain learning can provide more comprehensive EEG representations than a single pathway. However, their branches may primarily differ in convolutional scale, receptive field, auxiliary task, or feature-transformation strategy, and their outputs are generally integrated within a shared representation space.

More recent methods have further extended this direction through richer interaction mechanisms. MBBi-TCNet employs parallel branches, attention, and bidirectional temporal convolution to capture diverse temporal dependencies (Zhang et al., 2026). BioMIFormer introduces biologically inspired temporal, spatial, and spatiotemporal pathways with Transformer-based interaction and adaptive fusion (Liu et al., 2026). TFCA-TransNet combines raw temporal signals, Fourier-derived frequency information, spatial convolution, channel attention, and Transformer encoding (Miao et al., 2026). PLV-GCN + LSTM constructs functional-connectivity graphs using phase-locking values and combines graph convolution with recurrent temporal modeling (Kang et al., 2026). These approaches represent substantial progress toward temporal diversification, neurophysiologically inspired modeling, multimodal feature interaction, and graph-based connectivity learning. However, even when adaptive attention or fusion is employed, it does not necessarily provide an explicit, normalized, trial-specific allocation of importance among an adaptive time-frequency representation and a separate set of physiologically predefined rhythm representations.

Input-dependent feature modulation has also shown value beyond EEG decoding, including graph-guided contextual modeling, direction-aware attention, and adaptive processing of non-stationary signals (Yang et al., 2026; Chen et al., 2025; Peng et al., 2026). These studies support the broader principle that feature contributions can be adjusted according to individual inputs.

Despite this progress, two closely related challenges remain. First, data-driven frequency learning and physiologically predefined rhythm information are often used separately or merged into a common latent space, which may weaken their complementary roles. Second, existing attention and fusion mechanisms do not explicitly generate normalized trial-wise weights for semantically distinct temporal, learnable time-frequency, and rhythm-specific spectral branches. The key problem is therefore not merely to extract more features, but to preserve complementary representations and coordinate them according to individual EEG trials.

To address these challenges, we propose DMB-EDN, a Dynamic Multi-Branch EEG Decoding Network comprising a temporal branch, a learnable time-frequency branch, and a rhythm-specific spectral branch. The temporal branch captures local and long-range spatiotemporal dynamics through compact convolution, overlapping temporal windows, attention enhancement, and temporal convolution. The learnable time-frequency branch uses a Gabor-initialized filter bank to adapt its frequency responses, whereas the rhythm-specific spectral branch models physiologically meaningful EEG rhythms and inter-band relationships. Their trial-level representations are integrated through trial-conditioned dynamic fusion, which jointly processes the branch descriptors and generates softmax-normalized weights for each EEG trial. This mechanism enables input-dependent cooperation among complementary representations rather than relying on equal averaging, direct concatenation, or globally shared coefficients.

The main contributions of this study are summarized as follows:

(1) A multi-branch EEG representation framework is proposed to preserve complementary temporal, learnable time-frequency, and rhythm-specific spectral representations within distinct processing pathways.
(2) A trial-conditioned dynamic fusion strategy is developed to generate softmax-normalized branch weights and adaptively coordinate the three representations for each EEG trial.
(3) The proposed method is evaluated on two public EEG decoding datasets and one self-collected SCI dataset under subject-specific and leave-one-subject-out settings, providing benchmark comparisons and a preliminary offline clinical evaluation.

The remainder of this paper is organized as follows. Section 2 describes the proposed DMB-EDN architecture. Section 3 presents the datasets, experimental protocol, and comparative results. Section 4 reports the ablation and model analysis. Section 5 concludes the paper and discusses future work.

## 2 Methods

### 2.1 Overall network architecture

**Figure 1:**
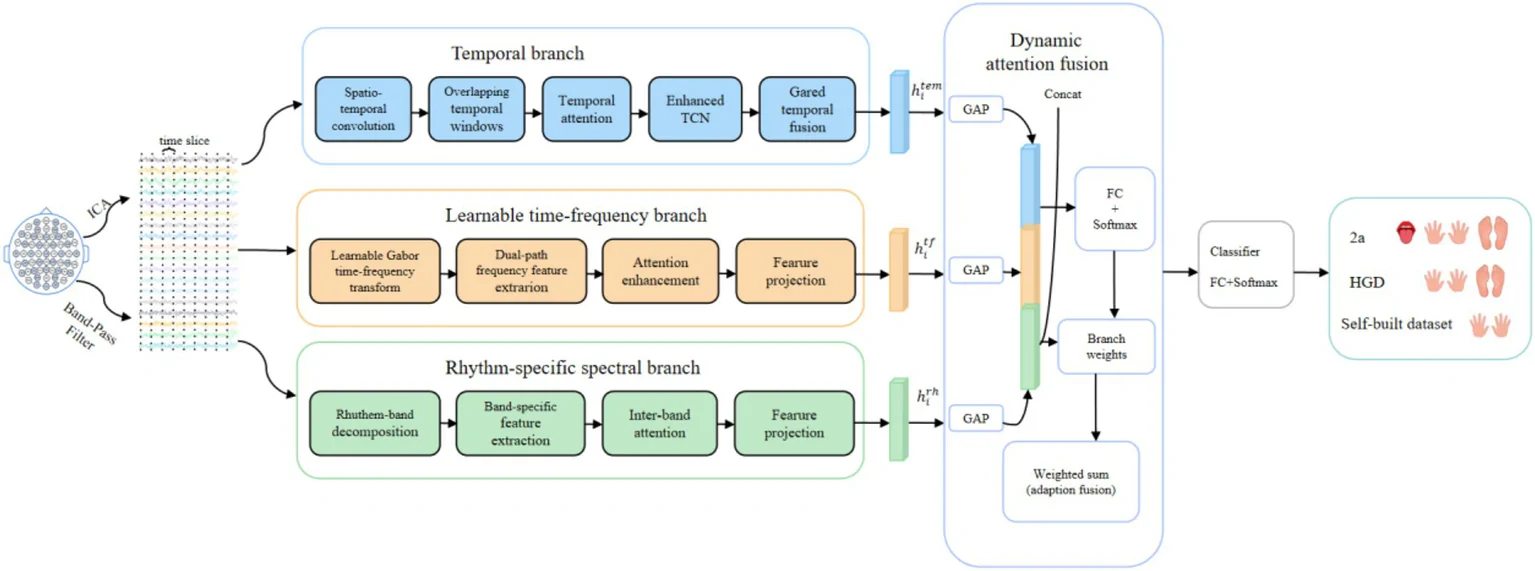
Architecture of the proposed DMB-EDN. EEG trials are processed by the temporal, learnable time-frequency, and rhythm-specific spectral branches to obtain complementary representations, which are subsequently coordinated through trial-conditioned dynamic fusion for classification. GAP denotes global average pooling, and FC denotes a fully connected layer.

The overall architecture of the proposed DMB-EDN is illustrated in Figure 1. The framework consists of three parallel representation branches: a temporal branch, a learnable time-frequency branch, and a rhythm-specific spectral branch. Their outputs are adaptively integrated through a sample-level dynamic fusion module. This design enables the network to capture complementary temporal and oscillatory information while accommodating trial-dependent variability and the non-stationary characteristics of EEG signals. The overall architecture is formulated in Equation 1 as

$$h_{i}=F_{\text{fusion}}(h_{i}^{\mathrm{tem}},h_{i}^{\mathrm{tf}},h_{i}^{\mathrm{rh}}),h_{i}^{b}=F_{b}(X_{i}),b\in B$$ (1)

Where $X_{i}$ denotes the i−th input EEG signal, B={tem,tf,rh} , and $h_{i}^{b}\in {\mathbb{R}}^{d}$ denotes the projected trial-level representation produced by branch b. The fused representation $h_{i}$ is subsequently used for classification.

Throughout this section, $H_{i}^{b}$ denotes the internal feature map of branch b, whereas $h_{i}^{b}$ denotes its projected trial-level representation. Temporal-window, rhythm-band, and cross-branch attention weights are denoted by $\beta_{i,m},\eta_{i,r},\text{and}\;\alpha_{i,b}$ , respectively. The classifier logits and predicted probabilities are represented by $z_{i}$ and $p_{i}$ .

MI-EEG contains complementary temporal and oscillatory information. The temporal branch captures waveform evolution, transient transitions, temporal ordering, and long-range dependencies. The learnable time-frequency branch uses parameterized Gabor filters to adapt temporally localized frequency responses, whereas the rhythm-specific spectral branch explicitly models canonical EEG rhythms and their inter-band relationships. The latter two branches provide complementary data-driven and physiology-guided descriptions of EEG oscillations rather than redundant frequency-domain representations.

### 2.2 Temporal branch

The temporal branch is designed to capture waveform evolution, transient changes, temporal ordering, and long-range dependencies directly from raw EEG sequences. It consists of a compact spatiotemporal convolutional encoder, overlapping temporal-window extraction, attention- enhanced temporal convolutional processing, and adaptive window-level fusion.

For the i−th EEG trial, the initial temporal feature sequence is obtained according to Equation 2:

$$H_{i}^{\mathrm{tem}}={ℰ}_{\mathrm{tem}}(X_{i})\in {\mathbb{R}}^{d_{t}\times T^{\prime }}$$ (2)

Where ${ℰ}_{\mathrm{tem}}(X_{i})$ denotes the spatiotemporal convolutional encoder, $T^{\prime }$ is the downsampled temporal length, and $d_{t}$ is the temporal feature dimension.

The encoder adopts depthwise separable convolution to learn compact channel-temporal representations. In the implementation, the first temporal convolution uses *F*_1_ = 16 filters with a kernel length of *L*_1_ = 64. With a depth multiplier of D = 2, the following convolutional block produces *F*_2_ = *F*_1_D = 32 feature maps. Batch normalization, ELU activation, average pooling, and dropout are employed to improve training stability and reduce overfitting.

**Figure 2:**
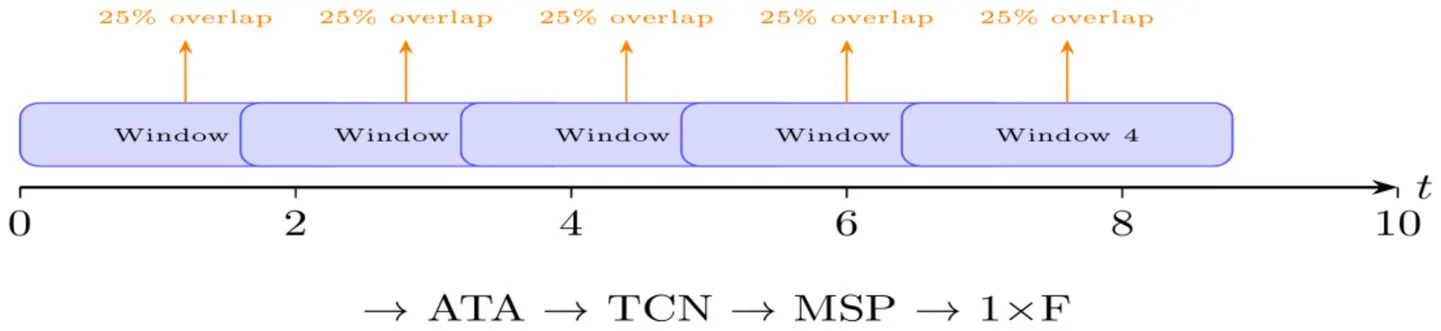
Overlapping sliding-window mechanism for temporal feature extraction. The feature sequence obtained from spatio-temporal convolution is divided into multiple overlapping windows at different temporal positions to capture multi-scale temporal dynamics.

To capture temporal patterns at different intervals, an overlapping sliding-window mechanism is applied to $H_{i}^{\mathrm{tem}}$ , as illustrated in Figure 2. The number of temporal windows is fixed at $N_{w}$ =5 across all datasets to maintain a consistent multi-window structure. Because the temporal input length differs among datasets, the window length and stride are determined according to the dataset-specific value of $T^{\prime }$ , rather than being fixed in seconds. Given an overlap ratio of ρ = 0.25, the window length is defined in Equation 3:

$$L_{w}=\lceil \frac{T^{\prime }}{1+(N_{w}-1)(1-\rho )}\rceil$$ (3)

and the corresponding window stride is calculated according to Equation 4:

$$S_{w}=L_{w}(1-\rho )$$ (4)

To ensure complete temporal coverage, the final window is aligned with the end of the feature sequence. The starting and ending positions of the m−th window are defined as in Equation 5:

$$s_{m}=\{\begin{array}{c} mS_{w},m=0,\ldots ,N_{w}-2\\ T^{\prime }-L_{w},\;m=N_{w}-1 \end{array},\;\;\;e_{m}=\min(T^{\prime },s_{m}+L_{w})$$ (5)

The corresponding window feature is extracted as according to Equation 6:

$$H_{i,m}^{\mathrm{tem}}=H_{i}^{\mathrm{tem}}[:,s_{m}:e_{m}]$$ (6)

Each window feature is then processed by an adaptive attention module and an enhanced temporal convolutional network , as formulated in Equation 7:

$$V_{i,m}^{\mathrm{tem}}=T_{\mathrm{tem}}(A_{\mathrm{tem}}(H_{i,m}^{\mathrm{tem}}))$$ (7)

Where $A_{\mathrm{tem}}$ denotes the temporal attention operation and $T_{\mathrm{tem}}$ denotes the enhanced temporal convolutional network.

The attention module can be configured as multi-head attention (Vaswani et al., 2017), squeeze-and-excitation attention (Hu et al., 2018), convolutional block attention (Woo et al., 2018), or adaptive attention, allowing the model to emphasize informative temporal or channel-wise responses.

**Figure 3:**
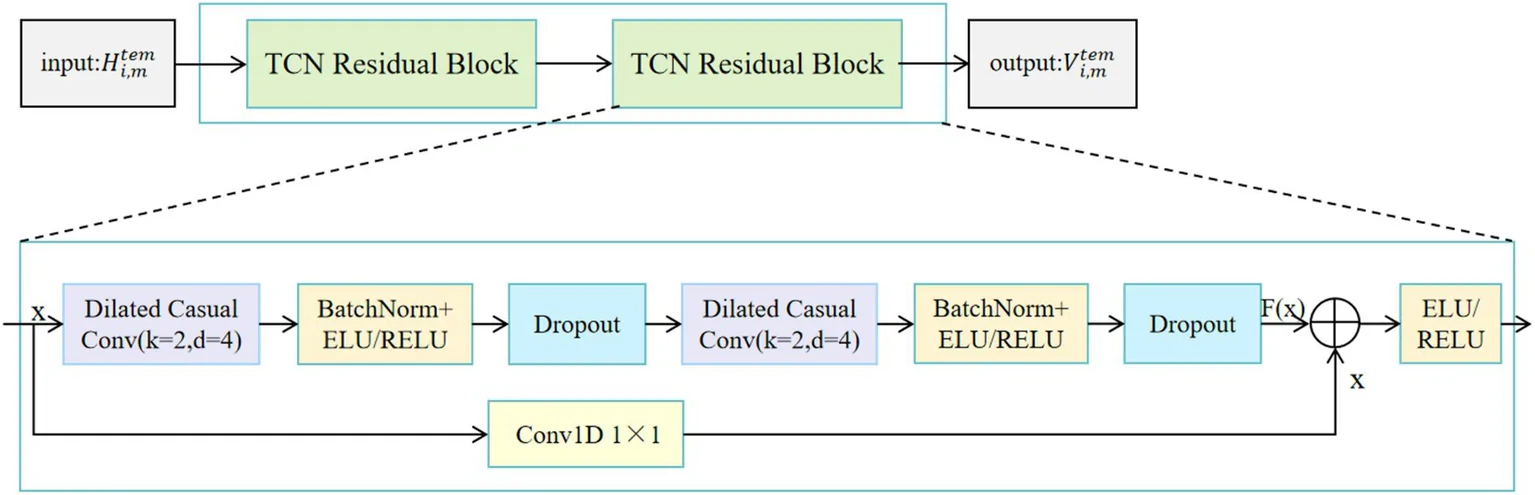
Architecture of the TCN composed of two residual blocks with different dilation rates. Each residual block employs dilated one-dimensional convolutions, batch normalization, ELU activation, and dropout operations to capture multi-scale temporal dependencies.

After attention enhancement, the Temporal Convolutional Network (TCN) further extracts hierarchical temporal dependencies through residual dilated convolutions, as shown in Figure 3. The dilation rate increases with network depth, enlarging the effective receptive field and enabling the model to capture both local and long-range temporal patterns.

**Figure 4:**
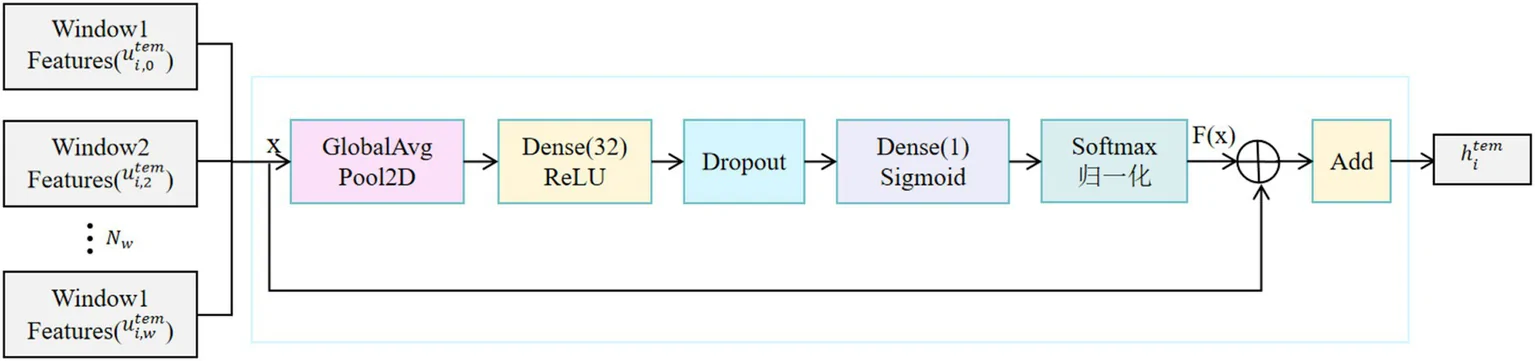
Adaptive window-level fusion for temporal feature integration. Global average pooling and shared fully connected layers are used to generate attention weights for different temporal windows, followed by adaptive weighted fusion to obtain the final temporal representation.

The processed window features are adaptively aggregated through the window-level fusion mechanism illustrated in Figure 4. Global average pooling is first applied to each processed window to obtain a compact descriptor, as defined in Equation 8:

$$u_{i,m}^{\mathrm{tem}}=\mathrm{GAP}(V_{i,m}^{\mathrm{tem}})$$ (8)

Where $u_{i,m}^{\mathrm{tem}}$ denotes the descriptor of the m−th temporal window.

A shared fully connected scoring function followed by softmax generates the normalized importance weight of each temporal window according to Equation 9:

$$\beta_{i,m}=\frac{\exp(a_{i,m}^{\mathrm{tem}})}{\sum_{m\prime =0}^{N_{w}-1}\exp(a_{i,m\prime }^{\mathrm{tem}})}$$ (9)

Where $a_{i,m}^{\mathrm{tem}}$ denotes the importance score assigned to the m−th temporal window. The softmax operation ensures that $\beta_{i,m}\geq 0$ and that the weights of all windows sum to one.

The final temporal branch representation is obtained through adaptive weighted aggregation, as defined in Equation 10:

$$h_{i}^{\mathrm{tem}}=\mathrm{tem}(\sum_{m=0}^{N_{w}-1}\beta_{i,m}u_{i,m}^{\mathrm{tem}})\in {\mathbb{R}}^{d}$$ (10)

Where tem denotes the temporal feature-projection layer, and d is the common feature dimension used for subsequent sample-level dynamic fusion.

Throughout the temporal branch, batch normalization and ELU activation improve optimization stability and nonlinear representation capability, whereas average pooling and dropout reduce overfitting while preserving informative temporal responses. The resulting representation $h_{i}^{\mathrm{tem}}$ integrates temporal information from multiple overlapping intervals and provides the temporal feature basis for subsequent sample-level dynamic fusion.

### 2.3 Learnable time-frequency branch

The learnable time-frequency branch is designed to extract discriminative and temporally localized oscillatory patterns from EEG signals. Unlike conventional time-frequency methods based on fixed basis functions, this branch employs a parameterized Gabor filter bank whose parameters are jointly optimized with the network. This design allows the time-frequency representation to adapt to task-relevant oscillatory patterns and subject- and trial-dependent frequency variations. Previous learnable wavelet-based EEG models have demonstrated the potential of parameterized time-frequency front ends for adaptive and interpretable EEG representation learning (Paillard et al., 2025).

For the q−th Gabor filter, the real and imaginary components are defined as in Equations 11 and 12, respectively:

gqRe(τ)=exp(−(τ−μq)22σq2)cos(2πfq(τ−μq)) (11)

gqIm(τ)=exp(−(τ−μq)22σq2)sin(2πfq(τ−μq)) (12)

Where $f_{q},\mu_{q},\text{and}\;\sigma_{q}$ denote the center frequency, temporal center, and bandwidth parameter of the q−th Gabor filter, respectively. Here, τ represents time in seconds; for a discrete sample index nnn, it is defined as $\tau_{n}=n/f_{s}$ , where $f_{s}$ is the sampling frequency.

The filter center frequencies are initialized over the 1–30 Hz range associated with commonly studied motor-related EEG activity. Importantly, this interval is used only for initialization rather than as a strict frequency constraint, allowing the filter parameters to adapt during end-to-end optimization.

For the i−th EEG trial $X_{i}\in {\mathbb{R}}^{C\times T}$ , the real and imaginary responses of the q−th Gabor filter are obtained through one-dimensional convolution. The corresponding amplitude response is calculated as according to Equation 13:

Ai,q=(Xi∗gqRe)2+(Xi∗gqIm)2+ε (13)

Where ∗ denotes one-dimensional convolution along the temporal dimension and ε>0 is a small constant introduced for numerical stability.

In the implementation, the mini-batch input is represented as $X\in {\mathbb{R}}^{B\times 1\times C\times T}$ , where B is the mini-batch size. Before applying the one-dimensional Gabor convolutions, the singleton dimension is removed, resulting in a tensor of size B×C×T . The amplitude responses generated by all Q filters are then stacked to form the learnable time-frequency feature map $H_{i}^{\mathrm{tf}}$ , where Q denotes the number of Gabor filters.

**Figure 5:**
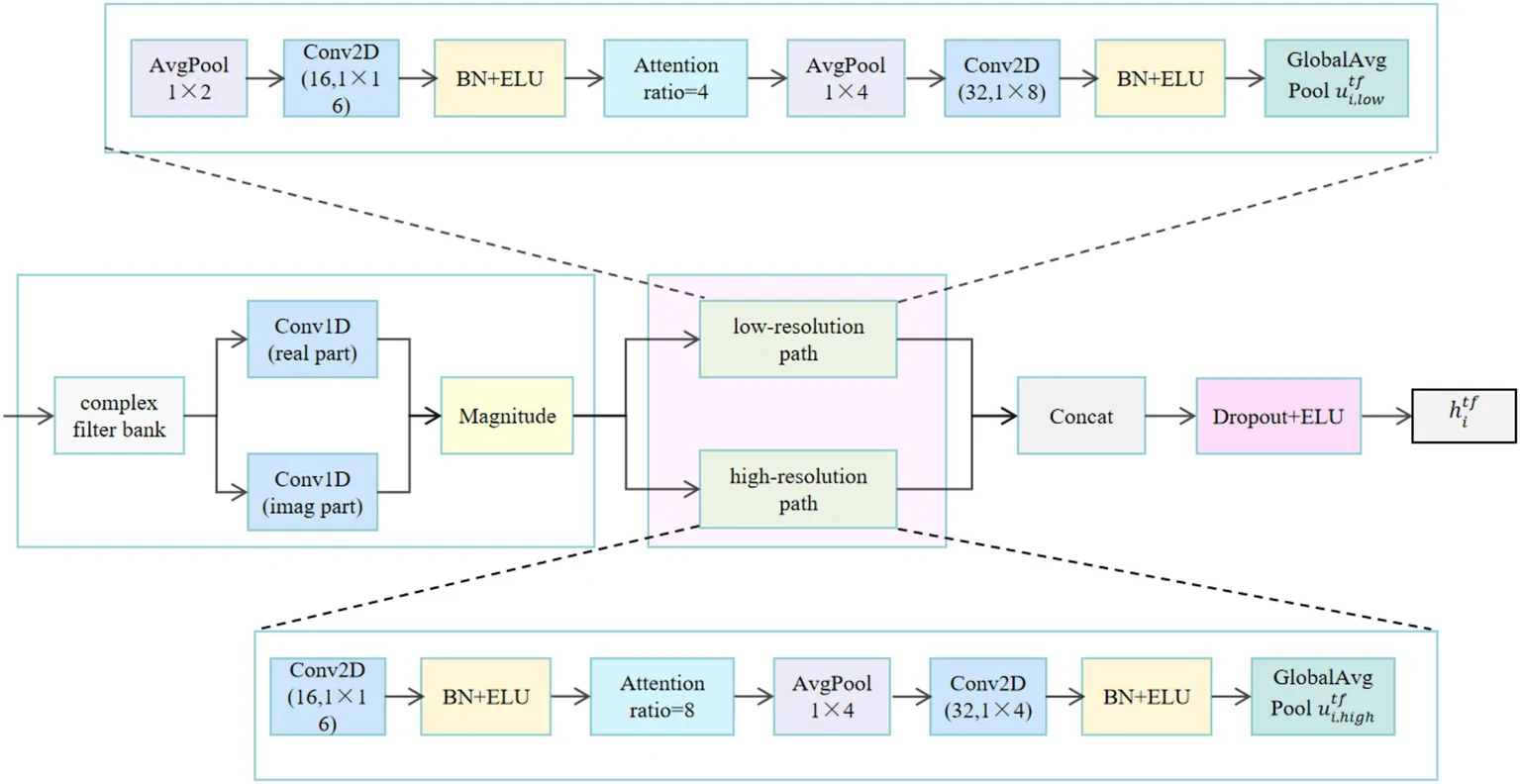
Architecture of the learnable Gabor filter bank and dual-path time-frequency branch. The input EEG signals are transformed into time-frequency representations and divided into high-frequency and low-frequency channels for complementary frequency feature extraction and fusion.

To capture oscillatory information at different resolutions, a dual-path multi-scale feature extraction module is applied to $H_{i}^{\mathrm{tf}}$ , as illustrated in Figure 5. The high-resolution path directly processes the learned feature map to preserve fine-grained and temporally localized oscillatory responses. In contrast, the low-resolution path first applies average pooling and then uses a larger effective receptive field to capture broader temporal–frequency contexts. Both paths are followed by feature encoding, attention enhancement, and global average pooling. The resulting descriptors are denoted by $u_{i,\text{high}}^{\mathrm{tf}}$ and $u_{i,\mathrm{low}}^{\mathrm{tf}}$ , respectively.

The final learnable time-frequency representation is obtained by concatenating and projecting the two descriptors, as defined in Equation 14:

$$\begin{array}{c} h_{i}^{\mathrm{tf}}=\mathrm{tf}(u_{i,\text{high}}^{\mathrm{tf}}\|u_{i,\mathrm{low}}^{\mathrm{tf}})\in {\mathbb{R}}^{d} \end{array}$$ (14)

Where ‖ denotes feature concatenation, tf denotes the time-frequency feature-projection layer, and d is the common feature dimension used for subsequent sample-level dynamic fusion.

The resulting representation $h_{i}^{\mathrm{tf}}$ combines fine-grained and broader oscillatory information. Unlike the rhythm-specific spectral branch, which is constrained by predefined EEG rhythm ranges, the learnable time-frequency branch provides an adaptive and data-driven representation of temporally localized frequency activity.

### 2.4 Rhythm-specific spectral branch

The rhythm-specific spectral branch is designed to model canonical neural oscillations associated with motor imagery. Unlike the learnable time-frequency branch, which adaptively learns temporally localized oscillatory patterns from data, this branch explicitly incorporates neurophysiological rhythm priors. Based on previous studies of event-related desynchronization and synchronization in sensorimotor rhythms (2), the input EEG signal is decomposed into four canonical frequency ranges: theta (4–8 Hz), alpha (8–13 Hz), beta (13–30 Hz), and gamma (30–45 Hz). This design preserves physiologically meaningful rhythm organization while allowing the model to learn trial-specific spectral representations.

Let ℛ={theta,,,alpha,,,beta,,,gamma} denotes the set of canonical EEG rhythms. For the i−th EEG trial $X_{i}\in {\mathbb{R}}^{C\times T}$ , the feature map associated with rhythm r is extracted as according to Equation 15:

$$\begin{array}{c} H_{i,r}^{\mathrm{rh}}={ℰ}_{r}(G_{r}(X_{i})),r\in ℛ \end{array}$$ (15)

Where $G_{r}$ denotes the differentiable rhythm-specific filtering operation and ${ℰ}_{r}$ denotes the corresponding feature encoder. The rhythm-specific filtering operations are configured to emphasize the target frequency range of each rhythm and are jointly optimized with the network.

Global average pooling is then applied to each rhythm-specific feature map to obtain the compact rhythm descriptor $u_{i,r}^{\mathrm{rh}}=\mathrm{GAP}(H_{i,r}^{\mathrm{rh}})$ . This band-specific processing preserves oscillatory characteristics associated with different canonical frequency ranges.

To adaptively estimate the relative contributions of different rhythms, a rhythm-level attention mechanism is introduced. A shared scoring function first generates an attention score $a_{i,r}^{\mathrm{rh}}$ for each rhythm descriptor. The normalized attention weight assigned to rhythm r is calculated as according to Equation 16:

$$\begin{array}{c} \eta_{i,r}=\frac{\exp(a_{i,r}^{\mathrm{rh}})}{\sum_{r\prime \in ℛ}\exp(a_{i,r\prime }^{\mathrm{rh}})} \end{array}$$ (16)

Where $\eta_{i,r}$ denotes the trial-specific importance of rhythm r . The softmax operation ensures that $\eta_{i,r}\geq 0$ and that the attention weights of all rhythm bands sum to one. This mechanism enables the network to emphasize the rhythms that contribute most strongly to the classification of each EEG trial.

The final rhythm-specific spectral representation is obtained through weighted aggregation and feature projection, as defined in Equation 17:

$$\begin{array}{c} h_{i}^{\mathrm{rh}}=\mathrm{rh}(\sum_{r\in ℛ}\eta_{i,r}u_{i,r}^{\mathrm{rh}})\in {\mathbb{R}}^{d} \end{array}$$ (17)

Where rh denotes the rhythm-specific feature-projection layer, and d is the common feature dimension used for subsequent sample-level dynamic fusion.

The resulting representation $h_{i}^{\mathrm{rh}}$ preserves the neurophysiological interpretability of canonical EEG rhythms while adaptively emphasizing the most informative rhythm-specific activity for each trial. Therefore, the rhythm-specific spectral branch provides a physiology-guided complement to the temporal branch and the data-driven learnable time-frequency branch.

### 2.5 Sample-level dynamic fusion

The three branches of DMB-EDN extract complementary representations from the same EEG trial. However, their relative importance may vary across trials because of the non-stationary and trial-dependent characteristics of EEG signals. Therefore, a sample-level dynamic fusion module is introduced to adaptively determine the contribution of each branch for each EEG trial.

As defined in the preceding sections, the temporal, learnable time-frequency, and rhythm-specific spectral branches produce the d−dimensional representations $h_{i}^{\mathrm{tem}},h_{i}^{\mathrm{tf}},\text{and}\;h_{i}^{\mathrm{rh}}$ , respectively. These representations are concatenated to form a joint branch descriptor defined in Equation 18:

$$\begin{array}{c} q_{i}=h_{i}^{\mathrm{tem}}\|h_{i}^{\mathrm{tf}}\|\;h_{i}^{\mathrm{rh}}\in {\mathbb{R}}^{3d} \end{array}$$ (18)

Where ‖ denotes feature concatenation.

The joint descriptor is subsequently passed through a fully connected scoring layer followed by a softmax function to generate trial-specific branch weights, as defined in Equation 19:

$$\begin{array}{c} \alpha_{i}=\text{softmax}(W_{a}q_{i}+b_{a}) \end{array}$$ (19)

Where $W_{a}\in {\mathbb{R}}^{3\times 3d}$ and $b_{a}\in {\mathbb{R}}^{3}$ are learnable parameters. The resulting branch-weight vector is expressed in Equation 20 as

$$\begin{array}{c} \alpha_{i}={\left[\alpha_{i,\mathrm{tem}},\alpha_{i,\mathrm{tf}},\alpha_{i,\mathrm{rh}}\right]}^{T} \end{array}$$ (20)

Where $\alpha_{i,\mathrm{tem}},\alpha_{i,\mathrm{tf}},\text{and}\;\alpha_{i,\mathrm{rh}}$ denote the contributions of the temporal, learnable time-frequency, and rhythm-specific spectral branches, respectively. The softmax operation ensures that the three weights are nonnegative and sum to one.

The final fused representation is obtained through weighted aggregation defined in Equation 21:

$$\begin{array}{c} h_{i}=\sum_{b\in ℬ}\alpha_{i,b}h_{i}^{b}=\alpha_{i,\mathrm{tem}}h_{i}^{\mathrm{tem}}+\alpha_{i,\mathrm{tf}}h_{i}^{\mathrm{tf}}+\alpha_{i,\mathrm{rh}}h_{i}^{\mathrm{rh}} \end{array}$$ (21)

Where B={tem,tf,rh} denotes the set of branches, and $h_{i}\in {\mathbb{R}}^{d}$ is the fused representation subsequently passed to the classification head.

Unlike fixed fusion strategies, the proposed module generates a distinct set of branch weights for each EEG trial. It can therefore emphasize temporal dynamics, adaptive time-frequency responses, or canonical rhythm information according to the characteristics of the individual sample. The effectiveness of the proposed trial-conditioned fusion mechanism is further evaluated against several fixed and learnable fusion strategies in the ablation study.

### 2.6 Classification and training objective

After sample-level dynamic fusion, the fused trial-level representation $h_{i}\in {\mathbb{R}}^{d}$ is directly fed into a fully connected classification head. The classification logits for the iii-th EEG trial are calculated as in Equation 22.

$$\begin{array}{c} z_{i}=W_{c}h_{i}+b_{c}\in {\mathbb{R}}^{K} \end{array}$$ (22)

Where $W_{c}\in {\mathbb{R}}^{K\times d}$ and $b_{c}\in {\mathbb{R}}^{K}$ are learnable classifier parameters, and K denotes the number of task classes. In this study, K=4 for the BCI Competition IV 2a dataset and the High Gamma Dataset, whereas K=2 for the self-collected spinal cord injury dataset.

The predicted probability that the i−th EEG trial belongs to class k is computed using the softmax function, as defined in Equation 23:

$$\begin{array}{c} p_{i,k}=\frac{\exp(z_{i,k})}{\sum_{j=1}^{K}\exp(z_{i},j)},\;\;k=1,\ldots ,K \end{array}$$ (23)

Where $z_{i,k}$ denotes the k−th element of the logit vector $z_{i}$ , and $p_{i,k}$ denotes the corresponding predicted probability. The probability vector is denoted by $p_{i}={\left[p_{i,1},,\ldots ,,p_{i,K}\right]}^{T}$ , with $\sum_{k=1}^{K}p_{i,k}=1$ .

The network is trained end-to-end using the cross-entropy loss. For a mini-batch containing B EEG trials, the classification objective is defined as in Equation 24.

$$\begin{array}{c} {ℒ}_{\mathrm{CE}}=-\frac{1}{B}\sum_{i=1}^{B}\sum_{k=1}^{K}y_{i,k}\log(p_{i,k}+\varepsilon ) \end{array}$$ (24)

Where $y_{i,k}\in \{0,1\}$ denotes the one-hot encoded ground-truth label of the i−th trial for class k , and ε>0 is a small constant introduced for numerical stability.

During training, the temporal branch, learnable time-frequency branch, rhythm-specific spectral branch, sample-level dynamic fusion module, and classification head are jointly optimized through backpropagation. This end-to-end optimization enables DMB-EDN to learn complementary temporal and oscillatory representations under a unified supervised classification objective. During inference, the predicted class label is obtained as in Equation 25.

$$\begin{array}{c} \hat{y_{i}}=\text{argmax}p_{i,k} \end{array}$$ (25)

Therefore, DMB-EDN performs EEG classification by jointly learning temporal dynamics, adaptive time-frequency responses, rhythm-specific spectral information, and trial-dependent branch contributions.

## 3 Experiments and results analysis

### 3.1 Dataset description and preprocessing

#### 3.1.1 Datasets

To evaluate the proposed method under both benchmark and clinical conditions, experiments were conducted on two public EEG decoding datasets and one self-collected clinical motor imagery dataset. The BCI Competition IV 2a dataset (BCI IV-2a) was adopted as the primary benchmark dataset, the High Gamma Dataset (HGD) was used for additional public validation, and a self-collected spinal cord injury (SCI) dataset was employed to evaluate the preliminary clinical applicability of the proposed model.

BCI IV-2a Dataset: This dataset contains EEG recordings from nine subjects performing four motor imagery tasks, including left hand, right hand, feet, and tongue movements. The motor imagery period lasted 4 s, and EEG signals were recorded using 22 electrodes at a sampling rate of 250 Hz (Tangermann et al., 2012).

HGD: The dataset includes EEG recordings from 14 healthy subjects performing four tasks: left hand, right hand, feet, and rest. Each subject completed approximately 1,000 four-second trials divided into 13 runs. The training set consists of trials from all runs except the last two runs, while the remaining runs were used for testing. This dataset features a higher signal-to-noise ratio and more stable high-frequency activity characteristics, making it suitable for validating frequency-domain and spectral modeling capabilities (Schirrmeister et al., 2017).

Self-collected SCI Dataset: The SCI dataset was collected in collaboration with the Department of Rehabilitation, Affiliated Hospital of Shandong University of Traditional Chinese Medicine. EEG signals were recorded from nine patients using a Boruikang W4 saline-based EEG system at 1,000 Hz. Each participant completed 80 three-second motor imagery trials, comprising 40 left-foot and 40 right-foot trials. The cohort included seven males and two females aged 38–60 years, with a mean age of 49.0 ± 7.1 years. According to the ASIA Impairment Scale, one participant was classified as AIS A, three as AIS B, two as AIS C, and three as AIS D. One injury was complete and eight were incomplete, and the time since injury ranged from 1 to 24 months. Detailed participant characteristics are provided in Table 1. Owing to its limited sample size, substantial inter-subject heterogeneity, and complex clinical recording conditions, this dataset was used only for a preliminary offline evaluation of cross-subject decoding feasibility.

**Table 1:** Demographic and clinical characteristics of participants with spinal cord injury.

Patient	Sex	Age (years)	AIS grade	Time since injury(months)
A01	M	44	D	7
A02	M	52	A	24
A03	M	57	D	12
A04	F	50	C	7
A05	M	38	C	5
A06	M	49	B	2
A07	F	60	D	6
A08	M	50	B	24
A09	M	41	B	1

#### 3.1.2 EEG signal preprocessing

For BCI IV-2a, all 22 EEG channels were retained. Each trial was sampled at 250 Hz, and the task-related interval from 1.5 to 6.0 s was extracted, resulting in 1,125 time points per trial. Each input sample was therefore represented as $X_{i}\in {\mathbb{R}}^{22\times 1125}$ .

For HGD, trials containing absolute amplitudes greater than 800 μV within the 0–4 s artifact-screening interval were excluded. Forty-four channels covering the sensorimotor cortex were retained, and the signals were resampled to 250 Hz. Exponential running standardization was then applied, after which EEG epochs from −0.5 to 4.0 s relative to the event marker were extracted. Each trial was represented as $X_{i}\in {\mathbb{R}}^{44\times 1125}$ .

For the self-collected spinal cord injury dataset, continuous 64-channel EEG recordings were read from the original BDF files. According to the event markers, a 3-s segment following the onset of left- or right-side motor imagery was extracted from each trial. The signals were band-pass filtered between 0.5 and 100 Hz, notch-filtered at 50 Hz, and re-referenced using the reference electrode standardization technique. The data were subsequently downsampled from 1,000 to 100 Hz. Trials were rejected when the absolute amplitude of any channel exceeded 70 μV, resulting in samples of size $X_{i}\in {\mathbb{R}}^{64\times 300}$ .

### 3.2 Experimental setup and evaluation metrics

Subject-specific evaluation was conducted on the BCI IV-2a and HGD datasets, whereas leave-one-subject-out validation was used for the self-collected SCI dataset. Each experiment was repeated with five random seeds, and the mean performance was reported. All baseline methods were independently reimplemented and evaluated using the same preprocessing procedures, data partitions, training protocol, early-stopping criterion, and evaluation metrics. Hyperparameters were selected using only the training and validation sets.

The temporal branch used 16 filters with a kernel size of 64 and a depth multiplier of 2. Its TCN contained two residual blocks with 32 filters, a kernel size of 4, and dilation rates of 1 and 2, together with five overlapping temporal windows. The learnable time-frequency branch used 32 filters with a kernel size of 64 and a stride of 16. Branch outputs were projected into a shared 128-dimensional representation space. The model was trained for up to 200 epochs using Adam, with an initial learning rate of $1\times 10^{-3}$ , an L2 coefficient of $1\times 10^{-3}$ , a batch size of 32, cosine annealing, and early stopping with a patience of 20 epochs.

The model was implemented in Python 3.9 using TensorFlow 2.10 and Keras 2.10. Experiments were conducted on an AMD Ryzen 78845H CPU with 28 GB RAM under 64-bit Windows 11 without GPU acceleration. Single-trial inference latency was measured with a batch size of 1 using preprocessed inputs of 22×1125 , excluding EEG acquisition, preprocessing, disk input/output, model loading, and initialization.

#### 3.2.1 Data partitioning and leakage prevention

All datasets were partitioned at the original-trial level before normalization, data augmentation, and model-based temporal windowing. For the BCI IV-2a dataset, the official training session was divided into training and validation subsets using a stratified 80:20 split, while the official evaluation session was reserved exclusively for final testing. For the High Gamma Dataset, the predefined training and test partitions were retained, and the training data were further divided into training and validation subsets. For the self-collected spinal cord injury dataset, a nested leave-one-subject-out protocol was used: one patient was held out for testing, one of the remaining patients was used for validation, and the others were used for training.

All data-dependent preprocessing parameters were estimated using the training subset only and were applied unchanged to the validation and test subsets. Data augmentation, when enabled, was restricted to the training subset. The test data were not used for normalization, early stopping, checkpoint selection, or hyperparameter optimization.

The overlapping temporal windows in DMB-EDN were generated online from each complete trial during the forward pass and were not treated as independent samples. Therefore, all windows derived from the same trial remained within the same subset, as expressed in Equation 26, the training, validation, and test sets were mutually exclusive: ensuring that

$$\begin{array}{c} {Ι}_{\text{train}}\cap {Ι}_{\mathrm{val}}={Ι}_{\text{train}}\cap {Ι}_{\text{test}}={Ι}_{\mathrm{val}}\cap {Ι}_{\text{test}}=\emptyset \end{array}$$ (26)

The same partitions were used for DMB-EDN and all baseline models to ensure fair comparison.

#### 3.2.2 Statistical analysis

Model performance was evaluated using classification accuracy, Cohen’s kappa coefficient (κ), macro-averaged F1 score (Macro-F1), balanced accuracy (BAcc), and class-wise recall. Accuracy measures the overall proportion of correctly classified trials, whereas κ quantifies agreement between the predicted and true labels after accounting for chance agreement. Macro-F1 and BAcc assign equal importance to each class and are therefore useful for assessing performance under potential class imbalance.

Let $a_{\mathrm{ij}}$ denote the number of trials whose true class is iii and predicted class is j , and let C be the number of classes. Classification accuracy is defined as in Equation 27.

$$\begin{array}{c} A_{\mathrm{acc}}=\frac{\sum_{i=1}^{C}a_{\mathrm{ii}}}{\sum_{i=1}^{C}\sum_{j=1}^{C}a_{\mathrm{ij}}} \end{array}$$ (27)

The precision, recall,and F1 score of class i are calculated as according to Equation 28.

$$\begin{array}{c} P_{i}=\frac{a_{\mathrm{ii}}}{\sum_{k=1}^{C}a_{\mathrm{ki}}},R_{i}=\frac{a_{\mathrm{ii}}}{\sum_{j=1}^{C}a_{\mathrm{ij}}},F1_{i}=\frac{2P_{i}R_{i}}{P_{i}+R_{i}} \end{array}$$ (28)

and Macro-F1 and BAcc are defined as in Equation 29.

$$\begin{array}{c} \text{Macro}-F1=\frac{1}{C}\sum_{i=1}^{C}F1_{i},\;\;\;\;\text{BAcc}=\frac{1}{C}\sum_{i=1}^{C}R_{i} \end{array}$$ (29)

Cohen’s kappa coefficient and the expected agreement $P_{e}$ are defined as in Equation 30.

$$\begin{array}{c} \kappa =\frac{A_{\mathrm{acc}}-P_{e}}{1-P_{e}},P_{e}=\frac{\sum_{i=1}^{C}(\sum_{j=1}^{C}a_{\mathrm{ij}}\sum_{k=1}^{C}a_{\mathrm{ki}})}{{\left(\sum_{i=1}^{C}\sum_{j=1}^{C}a_{\mathrm{ij}}\right)}^{2}} \end{array}$$ (30)

The inter-subject standard deviation of a metric x is calculated as according to Equation 31.

$$\begin{array}{c} S=\sqrt{\frac{1}{N-1}\sum_{d=1}^{N}{\left(x_{d}-\bar{x}\right)}^{2}} \end{array}$$ (31)

Where $x_{d}$ is the classification accuracy of the d-th subject, and N is the number of subjects.

Statistical comparisons between DMB-EDN and ATCNet were performed at the subject level. Normality of the paired accuracy differences was assessed using the Shapiro–Wilk test. A two-sided paired t-test was applied when normality was not rejected; otherwise, the Wilcoxon signed-rank test was used. Exact paired permutation tests, 95% BCa bootstrap confidence intervals, and effect sizes were additionally reported, with *p*-values adjusted across the three datasets using the Holm procedure. A corrected *p* < 0.05 was considered statistically significant. Row-normalized confusion matrices and class-wise recall were used to analyze class-specific errors.

### 3.3 Comparative models

As shown in Table 2, to comprehensively evaluate the effectiveness of the proposed DMB-EDN model, several representative MI-EEG decoding methods were selected as baseline models, including EEGNet (Lawhern et al., 2018), ShallowConvNet (Schirrmeister et al., 2017), EEGTCNet (Ingolfsson et al., 2020), TCNet_Fusion (Musallam et al., 2021), MBEEG_SENet (Altuwaijri et al., 2022) and ATCNet (Altaheri et al., 2023). These models cover different architectural paradigms, including lightweight convolutional networks, temporal convolutional models, attention-based frameworks, and hybrid temporal-feature fusion methods.

**Table 2:** Architectural comparison between DMB-EDN and representative MI-EEG decoding models.

Model	Main representation	Architecture structure	Attention or temporal modeling	Fusion mechanism	Explicit rhythm prior	Trial-conditioned branch weighting
EEGNet	Temporal–spatial	Single branch	Depthwise separable convolution	Not applicable	No	No
ShallowConvNet	Temporal–spatial	Single branch	Temporal and spatial convolution	Not applicable	No	No
EEG-TCNet	Temporal–spatial	Single branch	TCN	Not applicable	No	No
TCNet_Fusion	Multi-level temporal	Single branch	TCN	Feature-level fusion	No	No
MBEEGSE	Multi-branch temporal–spatial	Multi-level	SE recalibration	Shared multi-branch fusion	No	No
ATCNet	Window-based temporal	Multi-branch	MHA + TCN	Window-level integration	No	No
DMB-EDN	Temporal+time-frequency+spectral	Three banches	Attention, TCN and inter-band	Trial-conditioned dynamic fusion	Yes	Yes

### 3.4 Results and discussion

#### 3.4.1 Main results on BCI IV 2a

As shown in Table 3, DMB-EDN achieved the best performance on BCI IV-2a, with an average accuracy of 96.41%, a Macro-F1 score of 96.45%, a BAcc of 96.45%, and a Cohen’s kappa of 0.952. It outperformed ATCNet and maintained accuracies above 91% for all nine subjects, indicating consistently strong within-subject decoding performance. This improvement may benefit from the complementary three-branch representations and trial-conditioned dynamic fusion.

**Table 3:** Subject-specific classification performance on the BCI IV 2a dataset in terms of mean accuracy, Macro-F1, balanced accuracy, and Cohen’s kappa.

Dataset	Subject	TCNet_Fusion	EEGTCNet	EEGNet	ShallowConvNet	MBEEG SENet	ATCNet	DMB-EDN
BCI IV 2a	1	71.88	71.53	79.17	74.31	80.21	86.11	95.83
	2	54.86	42.70	52.43	51.04	52.43	64.24	91.67
	3	86.11	87.15	84.38	82.64	86.46	92.01	98.26
	4	59.38	58.33	59.38	59.03	72.22	80.56	96.18
	5	68.06	67.71	57.99	59.03	34.03	71.53	95.14
	6	55.21	47.92	52.08	42.36	57.64	68.75	95.14
	7	81.25	71.53	82.64	80.21	85.42	95.49	98.96
	8	74.65	73.96	75.00	81.25	81.25	83.68	97.22
	9	77.43	70.49	75.69	77.78	74.31	87.50	99.31
	MAcc	69.87	65.70	68.75	67.52	69.33	81.10	96.41
	F1	69.87	65.80	68.76	67.21	69.67	81.05	96.45
	BAcc	69.88	65.85	68.75	67.21	69.63	81.11	96.45
	Kappa	0.598	0.543	0.583	0.567	0.591	0.748	0.952

Inter-subject variability remained evident: Subject 2 performed relatively poorly across multiple models, whereas Subject 9 approached ceiling-level accuracy. This shared pattern may reflect differences in signal quality, motor imagery strategy, task engagement, or sensorimotor-rhythm discriminability.

#### 3.4.2 Additional validation on HGD

As an additional public-dataset validation experiment, HGD was used to evaluate the stability of DMB-EDN under relatively high-quality recording conditions. As shown in Table 4, DMB-EDN achieved an average accuracy of 99.82%, a Macro-F1 score of 99.82%, a BAcc of 99.82%, and a Cohen’s kappa of 0.998. Its accuracy differed from that of ATCNet by only 0.01 percentage points, indicating essentially comparable performance. Because several baseline models already approached ceiling-level accuracy, the HGD results are interpreted as supplementary evidence that DMB-EDN remains stable and competitive under near-saturated conditions, rather than as evidence of a meaningful advantage.

**Table 4:** Additional subject-specific validation on the HGD dataset in terms of mean accuracy (%), Macro-F1, balanced accuracy, and Cohen’s kappa.

Dataset	Subject	TCNet_Fusion	EEGTCNet	EEGNet	ShallowConvNet	MBEEG SENet	ATCNet	DMB-EDN
HGD	1	100	96.24	99.86	100	100	100	100
	2	99.75	82.74	92.97	100	99.14	99.88	99.88
	3	98.75	93.64	95.23	100	99.55	100	100
	4	91.88	98.86	93.74	100	99.78	100	100
	5	93.12	96.38	97.91	100	99.86	99.85	100
	6	91.25	72.59	91.28	100	98.74	99.85	99.89
	7	91.82	84.19	94.01	100	98.80	99.43	99.88
	8	91.25	90.83	95.72	100	99.54	99.85	99.85
	9	96.25	93.75	96.59	100	99.20	99.89	99.77
	10	96.88	83.76	92.00	100	98.15	99.88	99.75
	11	76.88	75.88	85.57	100	93.41	99.43	98.64
	12	89.38	79.59	94.07	96.25	99.32	99.43	99.89
	13	91.19	78.88	88.25	100	98.75	100	100
	14	68.75	65.57	71.36	68.98	85.00	99.89	100
	MAcc	89.41	84.57	91.98	97.52	97.80	99.81	99.82
	F1	89.40	84.46	91.96	97.53	97.81	99.80	99.82
	BAcc	89.41	84.51	91.97	97.52	97.81	99.81	99.82
	Kappa	0.828	0.794	0.893	0.967	0.971	0.998	0.998

Although the 1–30 Hz interval was used only to initialize the learnable Gabor filters rather than as a strict frequency constraint, it primarily emphasizes conventional low-to-mid-frequency sensorimotor rhythms. Since HGD contains potentially informative higher-frequency activity, broader initialization ranges will be evaluated through a dedicated sensitivity analysis in future work.

#### 3.4.3 Clinical validation on self-collected SCI dataset

To provide a preliminary clinical evaluation, DMB-EDN was assessed on the self-collected SCI dataset using leave-one-subject-out validation. As shown in Table 5, it achieved an average accuracy of 85.00%, a Macro-F1 score of 84.99%, a BAcc of 85.00%, and a Cohen’s kappa of 0.700, outperforming the compared methods. Despite the limited sample size and substantial inter-subject variability, DMB-EDN maintained relatively stable offline cross-subject decoding performance. Nevertheless, these findings should be considered preliminary, and larger multicenter cohorts and prospective online BCI evaluations are required before clinical applicability can be established.

**Table 5:** LOSO clinical validation on the self-collected spinal cord injury dataset in terms of mean accuracy, Macro-F1, balanced accuracy, and Cohen’s kappa.

Dataset	Subject	TCNet_Fusion	EEGTCNet	EEGNet	ShallowConvNet	MBEEG SENet	ATCNet	DMB-EDN
SCI	1	83.75	65.00	66.25	73.75	71.25	76.25	96.25
	2	67.50	55.00	71.25	70.00	82.50	75.00	91.25
	3	75.00	50.00	63.75	51.25	55.00	70.00	78.75
	4	71.25	50.00	67.50	73.75	82.50	85.00	90.00
	5	78.75	51.25	65.00	72.50	50.00	73.75	93.75
	6	47.50	57.50	61.25	45.00	62.50	76.25	92.50
	7	57.50	46.25	52.50	62.50	76.25	76.25	65.00
	8	71.25	72.50	47.50	66.25	50.00	78.75	77.50
	9	57.50	56.25	65.00	71.25	50.00	65.00	80.00
	MAcc	67.78	55.97	62.22	65.25	64.44	75.13	85.00
	F1	67.53	49.89	61.69	64.99	64.35	75.10	84.99
	BAcc	68.00	56.05	62.00	65.00	64.50	75.08	85.00
	Kappa	0.356	0.119	0.244	0.303	0.289	0.552	0.700

**Figure 6:**
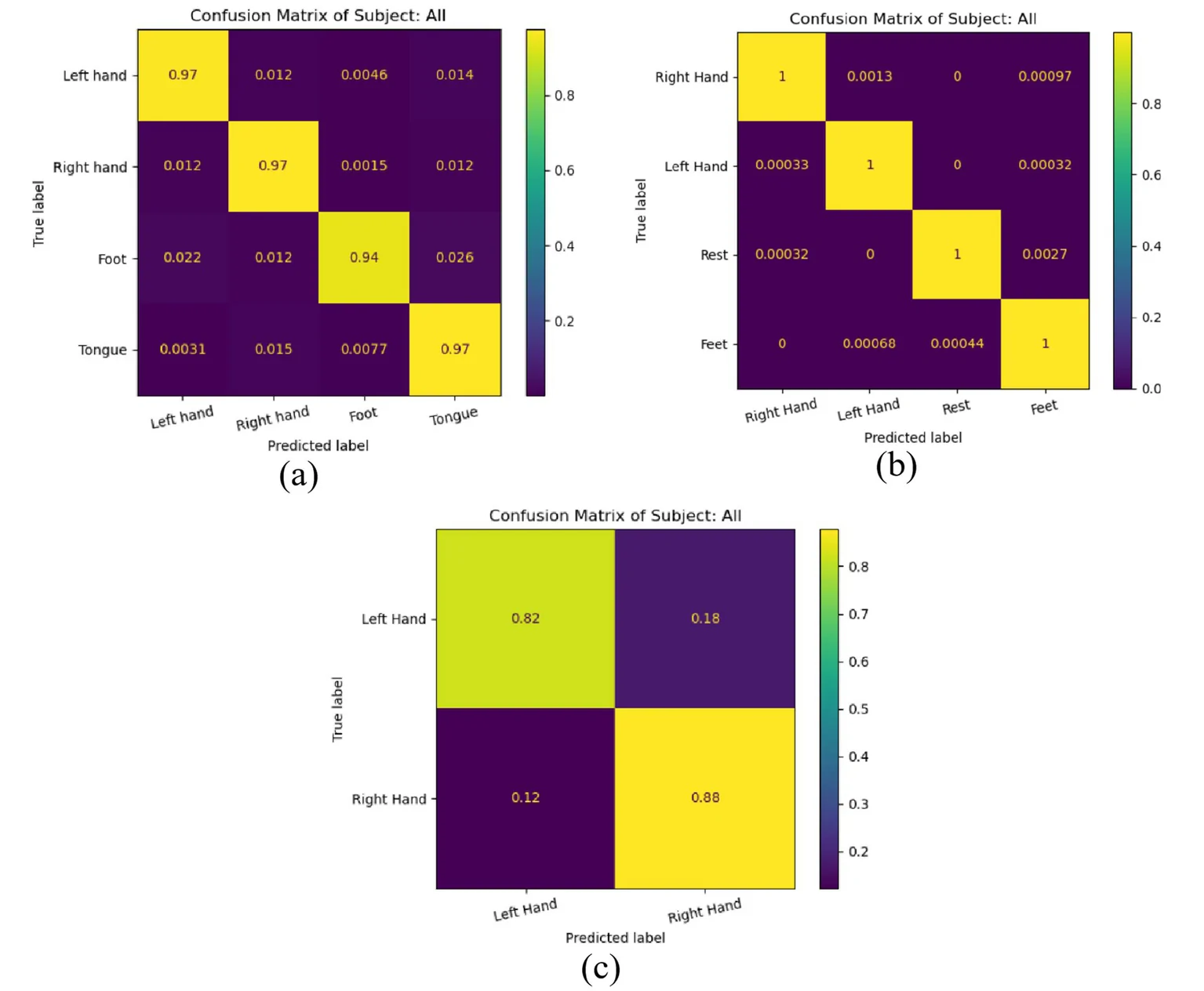
Average confusion matrices of DMB-EDN on each dataset. **(a)** BCI Competition IV 2a Dataset; **(b)** High Gamma Dataset; **(c)** Self-collected clinical dataset. Diagonal elements represent the classification accuracy for each class, and off-diagonal elements represent the misclassification rate.

Figure 6 presents the average row-normalized confusion matrices of DMB-EDN on the three datasets, together with the corresponding class-wise recall values. On BCI IV-2a, all motor imagery classes achieved high recognition rates, although relatively greater confusion was observed between foot and tongue imagery. On HGD, the class-wise recall values approached ceiling level, indicating stable decoding performance under near-saturated conditions. In contrast, the self-collected SCI dataset exhibited more pronounced inter-class confusion, particularly between left-hand and right-hand imagery. This pattern may be associated with stronger noise contamination, greater inter-subject variability, and less discriminative sensorimotor patterns in the clinical recordings. Nevertheless, DMB-EDN maintained relatively balanced class-wise performance, as reflected by the normalized confusion matrix, macro-F1 score, and balanced accuracy.

#### 3.4.4 Subject-level statistical comparison with ATCNet

As shown in Table 6, DMB-EDN improved the mean accuracy on BCI IV-2a by 15.32 percentage points. The improvement remained significant after Holm correction (p=0.0023) and was supported by the permutation analysis $\left(p_{\text{Holm}}=0.0117\right)$ , with a large effect size $\left(d_{z}=1.76\right)$ .

**Table 6:** Subject-level statistical comparison between DMB-EDN and ATCNet across the three datasets.

Dataset	Mean difference (pp)	95% CI (pp)	Shapiro–Wilk p	Primary test	Holm-adjusted p	Permutation $p_{\text{Holm}}$	Effect size
BCI IV 2a	+15.32	[+10.15, +21.10]	0.4667	Paired t-test	0.0023	0.0117	$d_{z}$ =1.76,large
HGD	+0.01	[−0.18, +0.13]	0.0051	Wilcoxon	0.6744	0.8906	$r_{\mathrm{rb}}$ =0.17,small
Self-collected SCI	+9.86	[+1.67, +15.28]	0.1496	Paired t-test	0.0480	0.0625	$d_{z}$ =0.93,large

On HGD, the mean difference was only 0.01 percentage points, and the confidence interval included zero. Neither the Wilcoxon test nor the permutation test indicated a significant difference, suggesting essentially comparable performance between DMB-EDN and ATCNet under the near-saturated conditions of this dataset.

On the self-collected SCI dataset, DMB-EDN achieved a mean improvement of 9.86 percentage points with a large effect size $\left(d_{z}=0.93\right)$ . Although the paired t−test remained marginally significant after Holm correction (p=0.0480) , the corrected permutation result did not reach significance $\left(p_{\text{Holm}}=0.0625\right)$ . Therefore, this result is interpreted as preliminary evidence of improved offline decoding performance rather than definitive evidence of clinical superiority.

#### 3.4.5 Computational complexity and offline inference efficiency

For practical MI-BCI applications, decoding performance should be considered together with computational complexity and inference efficiency. Representative baseline models and DMB-EDN were therefore compared in terms of trainable parameters, FLOPs, and single-trial inference time using the same BCI IV-2a input setting of 22 channels and 1,125 time points. All measurements were performed on the CPU-only workstation described in Section 3.2 with a batch size of 1.

As shown in Table 7, EEGNet and EEGTCNet have smaller parameter counts and lower computational costs, whereas DMB-EDN achieved the highest classification accuracy at the expense of increased complexity. DMB-EDN contains 2.55 million trainable parameters, requires 185.62 MFLOPs per forward pass, and has an average CPU inference latency of 142.41 ms per trial. The additional cost mainly arises from its three parallel representation branches and trial-conditioned dynamic fusion.

**Table 7:** Computational complexity comparison of DMB-EDN and baseline models.

Model	Parameters (M)	FLOPs(M)	Inference time (ms/trial)	Accuracy on BCI IV 2a (%)	Remarks
EEGNet	0.0026	26.30	11.25	68.75	Lightweight baseline
ShallowConvNet	0.0474	127.20	9.04	67.52	Classical CNN baseline
EEGTCNet	0.0043	13.72	19.46	65.70	Temporal convolution baseline
ATCNet	0.1152	59.58	79.46	81.10	Strong attention-TCN baseline
DMB-EDN	2.5500	185.62	142.41	96.41	Full proposed model

Parameters, FLOPs, and inference time were measured using the same BCI IV 2a input setting. Inference time was measured with batch size 1 on the same CPU platform without GPU acceleration.

The reported latency included only model inference on preprocessed trials already loaded into memory; EEG acquisition, preprocessing, disk input/output, model loading, and initialization were excluded. Therefore, it does not represent end-to-end online BCI latency. Practical deployment would additionally involve signal acquisition, buffering, preprocessing, decision generation, and feedback delivery. Memory, power, thermal, and latency constraints may also limit deployment on low-power embedded devices. Thus, embedded and real-time use remains to be validated through model compression, hardware-aware optimization, embedded-platform testing, and prospective online closed-loop BCI experiments.

#### 3.4.6 Feature distribution visualization

To examine the learned feature distributions, t-SNE was applied to the trial-level representations extracted before the final classification layer on the BCI IV-2a dataset. The analysis was conducted separately for each subject using all test trials. Features were standardized and reduced to at most 50 dimensions using PCA before projection. Identical samples and t-SNE settings were used for all models, with a perplexity of 20, a learning rate of 200, 2,000 iterations, PCA initialization, and a fixed random seed of 42.

**Figure 7:**
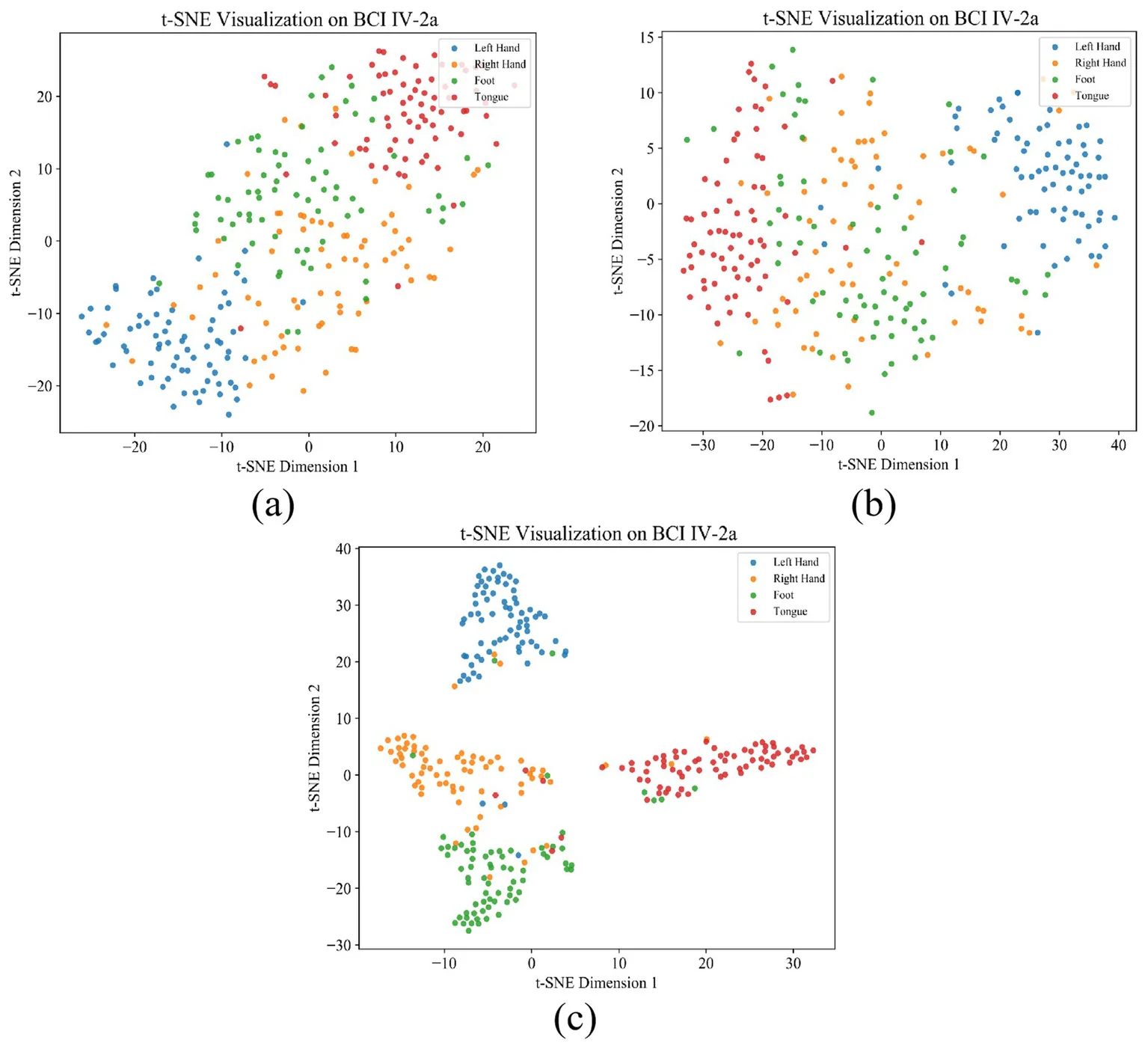
t-SNE visualization of feature representations on the BCI IV-2a dataset **(a)** EEGNet; **(b)** ATCNet; **(c)** DMB-EDN. Different colors represent different motor imagery classes: Left Hand, Right Hand, Foot, and Tongue.

As shown in Figure 7, EEGNet exhibits relatively dispersed representations with considerable class overlap, whereas ATCNet produces a more structured distribution. DMB-EDN shows comparatively compact within-class clusters and reduced overlap between several motor imagery classes, suggesting that its complementary representation branches and trial-conditioned dynamic fusion contribute to a more discriminative feature space. Nevertheless, because t-SNE is sensitive to projection settings and does not fully preserve global relationships, the visualization is considered qualitative evidence only.

For quantitative evaluation, the Davies–Bouldin index was calculated from the original high-dimensional representations before t-SNE projection. DMB-EDN achieved the lowest value of 1.08 ± 0.12, compared with 1.41 ± 0.15 for ATCNet, 1.54 ± 0.16 for TCNet-Fusion, 1.68 ± 0.18 for EEG-TCNet, and 1.92 ± 0.21 for EEGNet. The lower index indicates improved intra-class compactness and inter-class separation, providing quantitative support for the patterns observed in Figure 7.

#### 3.4.7 Analysis of trial-conditioned fusion weights

**Figure 8:**
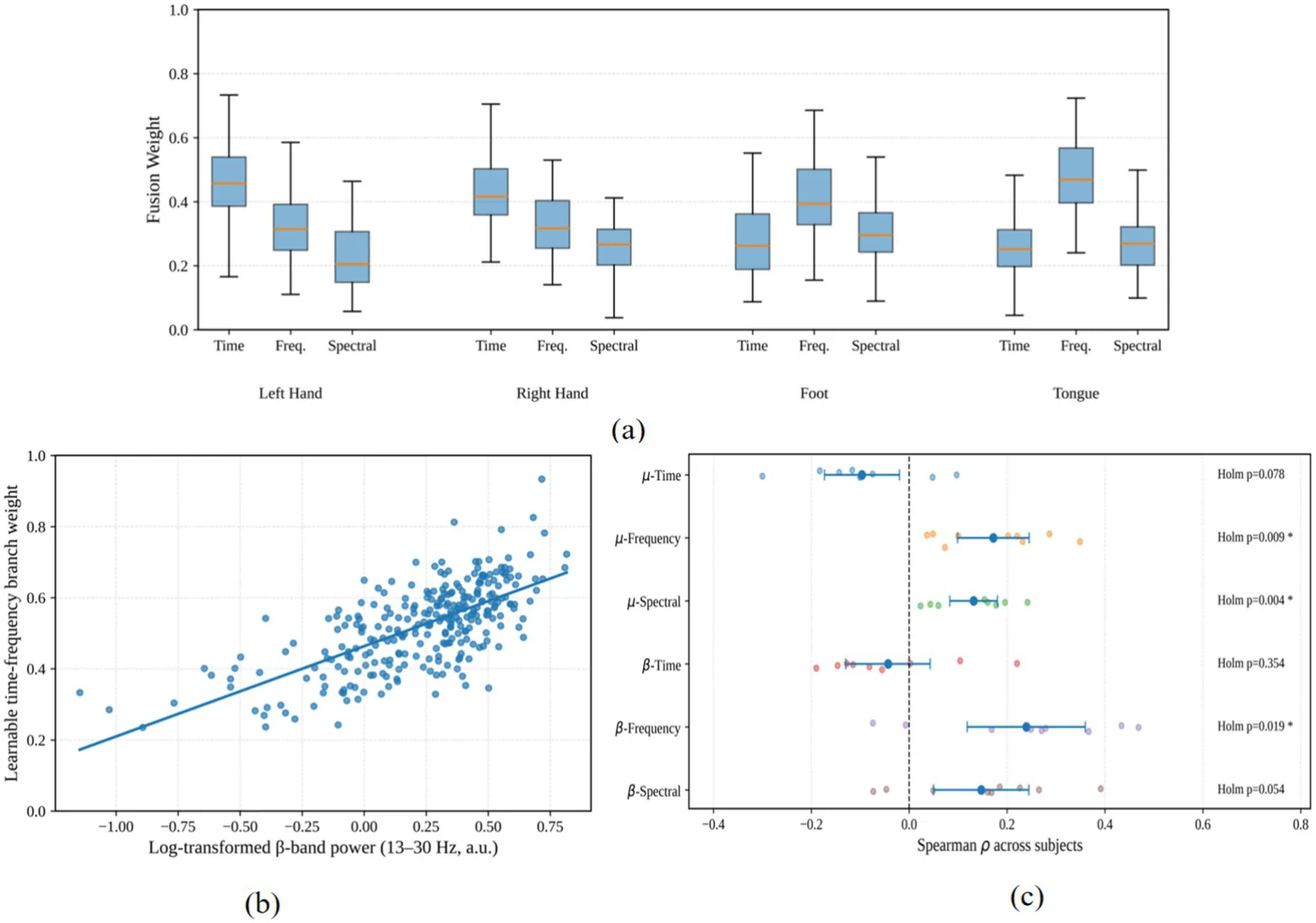
Trial-conditioned fusion-weight analysis on the BCI IV-2a dataset. **(a)** Class-wise distributions of the three branch weights. **(b)** Relationship between β-band power and the learnable time-frequency branch weight for Subject 5. **(c)** Across-subject Spearman correlations between sensorimotor-rhythm power and branch weights. Asterisks indicate significance after Holm correction.

To examine the adaptive behavior of trial-conditioned dynamic fusion, the softmax-normalized weights assigned to the temporal, learnable time-frequency, and rhythm-specific spectral branches were extracted from all test trials on BCI IV-2a. The analyses were first conducted within each subject and then summarized across subjects. As shown in Figure 8a, the temporal branch received relatively higher weights for left- and right-hand imagery, whereas the learnable time-frequency branch contributed more strongly to foot and tongue imagery. The rhythm-specific spectral branch showed a lower average contribution but retained trial-to-trial variability. These class-dependent shifts and within-class variations indicate that the fusion module does not reduce to fixed or globally shared branch coefficients.

Figure 8b shows a representative positive association between log-transformed β-band power and the learnable time-frequency branch weight for Subject 5 (ρ = 0.66, *p* < 0.001). As shown in Figure 8c, the group-level summary of within-subject Spearman correlations further revealed significant associations between μ-band power and the learnable time-frequency and rhythm-specific spectral branch weights ( $p_{\text{Holm}}=0.009\;$ and 0.004), and between β-band power and the learnable time-frequency branch weight ( $p_{\text{Holm}}=0.019\;$ ). The β-band association with the rhythm-specific spectral branch did not remain significant after correction ( $p_{\text{Holm}}=0.054$ ). These results suggest that the model adaptively reallocates branch contributions according to trial-specific oscillatory characteristics. Because the softmax weights sum to one, they should be interpreted as relative model-based contributions rather than direct neurophysiological biomarkers.

## 4 Design and analysis of ablation experiments

To further analyze the effectiveness of different modules in DMB-EDN, ablation experiments were conducted on the BCI IV-2a dataset. The experiments focused on three aspects: branch-level architecture, component-level mechanisms, and attention-module selection. All experiments were performed under the same training strategy, preprocessing pipeline, and evaluation settings to ensure fair comparison.

### 4.1 Branch-level ablation experiments

To systematically evaluate the individual contributions and complementarity of the three representation branches, seven configurations (M1–M7) were constructed, covering all non-empty combinations of the temporal, learnable time-frequency, and rhythm-specific spectral branches. The results are presented in Table 8.

**Table 8:** Performance comparison of branch-level ablation experiments on the BCI IV-2a dataset.

Model configuration	Accuracy (%)	Kappa	Number of parameters (M)	Accuracy difference (pp)
M1(T)	92.18 ± 1.42	0.896 ± 0.019	0.85	−4.23
M2(TF)	90.56 ± 1.68	0.874 ± 0.022	1.12	−5.85
M3(RS)	89.23 ± 1.85	0.857 ± 0.025	0.68	−7.18
M4(T + TF)	94.67 ± 1.18	0.929 ± 0.016	1.97	−1.74
M5(T + RS)	93.82 ± 1.26	0.917 ± 0.017	1.53	−2.59
M6(TF + RS)	93.16 ± 1.34	0.909 ± 0.018	1.80	−3.25
M7(T + TF + RS)	96.41 ± 0.85	0.952 ± 0.012	2.55	0.00

T, temporal branch; TF, learnable time-frequency branch; RS, rhythm-specific spectral branch; pp, percentage points.

Among the single-branch configurations, the temporal branch (M1) achieved the highest accuracy of 92.18%, indicating that temporal dynamics provide the strongest standalone representation. Among the dual-branch models, the combination of the temporal and learnable time-frequency branches (M4) performed best, achieving 94.67% accuracy and exceeding M1 by 2.49 percentage points. The learnable time-frequency and rhythm-specific spectral combination (M6) achieved 93.16%, outperforming the corresponding single-branch models by 2.60 and 3.93 percentage points, respectively, which supports their complementary rather than redundant roles. The full three-branch model (M7) obtained the best performance, with an accuracy of 96.41% and a Cohen’s κ of 0.952, exceeding the strongest dual-branch configuration by 1.74 percentage points. These results confirm that the three branches provide distinct and complementary information for EEG decoding.

### 4.2 Component-level ablation experiments

Based on the full three-branch architecture, several key modules were further removed or replaced to analyze their individual contributions. The results are shown in Table 9.

**Table 9:** Performance of component-level ablation experiments on the BCI IV-2a dataset.

Model configuration	Accuracy (%)	Kappa	Accuracy difference (pp)
C1(w/o Attn)	94.23 ± 1.18	0.923 ± 0.016	−2.18
C2(Single-window)	94.78 ± 1.10	0.930 ± 0.015	−1.63
C3(w/o LTF)	95.34 ± 1.02	0.938 ± 0.014	−1.07
C4(w/o IBA)	95.06 ± 1.05	0.934 ± 0.014	−1.35
C5(Full)	96.41 ± 0.85	0.952 ± 0.012	0.00

Attn, attention enhancement; LTF, learnable time-frequency transformation; IBA, inter-band attention; pp, percentage points.

Removing the attention mechanism (C1) caused the largest performance decrease, reducing the accuracy from 96.41 to 94.23%. This result indicates that attention-based feature enhancement plays an important role in emphasizing discriminative EEG patterns. Replacing the multi-scale temporal window mechanism with a single-window configuration (C2) reduced the accuracy to 94.78%, suggesting that multi-scale temporal modeling helps capture EEG dynamics at different temporal resolutions. In addition, removing the learnable time-frequency transformation (C3) and the inter-band attention mechanism (C4) both led to noticeable performance degradation. These results suggest that adaptive frequency representation learning and cross-band interaction modeling are both beneficial for MI-EEG decoding. Overall, the ablation experiments demonstrate that the performance improvement of DMB-EDN is achieved through the joint contribution of multi-domain representation learning, attention enhancement, adaptive fusion, and multi-scale temporal modeling.

### 4.3 Comparison of fusion strategies

To further investigate the contribution of the proposed trial-conditioned fusion mechanism, five multi-branch fusion strategies were evaluated under the same experimental protocol, as summarized in Table 10. The same temporal, learnable time-frequency, and rhythm-specific spectral branch encoders were retained in all configurations, and only the fusion mechanism was changed.

**Table 10:** Comparison of different multi-branch fusion strategies on the BCI competition IV 2a dataset.

Configuration	Trial-conditioned	Weight assignment	Accuracy (%)	Kappa	Parameters (M)	Accuracy decrease (pp)
F1(Avg)	No	Fixed (1/3) for each branch	94.68 ± 1.14	0.929 ± 0.015	2.51	−1.73
F2(Concat)	No	No explicit branch weights	95.24 ± 1.02	0.936 ± 0.014	2.68	−1.17
F3(Fixed)	No	Predefined shared weights	95.12 ± 1.06	0.935 ± 0.014	2.51	−1.29
F4(Global)	No	Trainable weights shared across all trials	95.57 ± 0.96	0.941 ± 0.013	2.51	−0.84
F5(Dynamic)	Yes	Trial-specific softmax weights	96.41 ± 0.85	0.952 ± 0.012	2.55	0.00

Avg, equal averaging; Concat, feature concatenation; Fixed, predefined fixed-weight fusion; Global, globally learned shared weighting; Dynamic, trial-conditioned dynamic fusion; pp, percentage points.

Equal averaging (F1) produced the largest performance decrease, reducing the accuracy from 96.41 to 94.68%. This indicates that assigning identical contributions to all three branches cannot adequately reflect their different relevance to individual EEG trials. Feature concatenation (F2) improved the accuracy to 95.24% by preserving the complete branch representations, but increased the fused feature dimension and model size. Fixed-weight fusion (F3) achieved an accuracy of 95.12%, suggesting that predefined, trial-independent weights cannot adequately accommodate EEG non-stationarity. Globally learned weighting (F4) further improved the accuracy to 95.57%, indicating that the three branches have different overall contributions to EEG decoding. However, because one learned weight vector was shared across all trials, this method could not account for trial-specific variations. In contrast, the proposed trial-conditioned dynamic fusion strategy (F5) generated input-dependent softmax weights for each trial and achieved the best performance, with an accuracy of 96.41% and a Cohen’s kappa coefficient of 0.952. It improved accuracy by 0.84 percentage points over globally learned weighting while introducing only a limited parameter increase. These results indicate that the benefit of DMB-EDN arises not only from combining complementary representations but also from dynamically coordinating their contributions at the individual-trial level.

### 4.4 Ablation experiments on attention mechanism types

To further analyze the influence of different attention mechanisms, five configurations (A1-A5) were evaluated, and the corresponding results are shown in Table 11.

**Table 11:** Performance comparison of attention mechanism ablation experiments on the BCI IV-2a dataset.

Attention type	Accuracy (%)	Kappa	Increase in parameters (%)	Increase in inference time (%)
A1 (None)	94.23 ± 1.18	0.923 ± 0.016	0.00	0.00
A2 (SE)	95.12 ± 1.06	0.935 ± 0.014	0.12	1.2
A3(CBAM)	95.45 ± 1.01	0.939 ± 0.013	0.18	2.1
A4 (MHA)	95.78 ± 0.95	0.944 ± 0.013	0.32	3.8
A5(Adaptive)	96.41 ± 0.85	0.952 ± 0.012	0.35	4.2

SE, squeeze-and-excitation; CBAM, convolutional block attention module; MHA, multi-head attention. Parameter and inference-time increases are calculated relative to A1.

The results show that all attention mechanisms improve the decoding performance compared with the model without attention. Among the fixed attention modules, multi-head attention (MHA) achieved better performance than SE and CBAM. The proposed adaptive attention strategy (A5) achieved the best overall performance, with an accuracy of 96.41% and a Kappa coefficient of 0.952. Compared with the model without attention, the adaptive attention mechanism improved the accuracy by 2.18 percentage points while introducing only a small increase in parameters and inference time. These findings indicate that the adaptive attention strategy provides a more effective trade-off between decoding performance and computational overhead under the evaluated setting.

## 5 Conclusion

This paper proposed DMB-EDN, a dynamic multi-branch network that jointly learns temporal dynamics, adaptive time-frequency patterns, and rhythm-specific spectral information for motor imagery EEG decoding. These complementary representations are integrated through trial-conditioned dynamic fusion. DMB-EDN achieved strong subject-specific performance on the BCI Competition IV 2a dataset and performance comparable to the strongest baseline on the High Gamma Dataset. Evaluation on the self-collected spinal cord injury dataset provided preliminary evidence of offline feasibility in a small clinical cohort. Ablation studies further confirmed the complementary contributions of the three representation branches and dynamic fusion. Nevertheless, the multi-branch architecture remains more computationally demanding than lightweight EEG models, and the reported processing latency does not establish end-to-end real-time BCI capability. Memory, power, and latency constraints may also limit direct deployment on low-power embedded devices. Future work will investigate model compression, hardware-aware optimization, cross-subject and cross-domain generalization, neurophysiological interpretability, larger independent multicenter clinical validation, and prospective online closed-loop BCI evaluation.

## Data Availability

The BCI competition IV 2a dataset and high gamma dataset are publicly available. The self-collected spinal cord injury dataset is not publicly available due to patient privacy and ethical restrictions imposed by the Ethics Committee of the Affiliated Hospital of Shandong University of Traditional Chinese Medicine. Requests to access the datasets should be directed to 15254717805@163.com (J. Cai).
